# The Effect of Treadmill Walking on Gait and Upper Trunk through Linear and Nonlinear Analysis Methods

**DOI:** 10.3390/s19092204

**Published:** 2019-05-13

**Authors:** Liang Shi, Feng Duan, Yikang Yang, Zhe Sun

**Affiliations:** 1 College of Artificial Intelligence, Nankai University, Tianjin 300350, China; liangshi@mail.nankai.edu.cn (L.S.); yangyikang@mail.nankai.edu.cn (Y.Y.); 2 Computational Engineering Applications Unit, Head Office for Information Systems and Cybersecurity, RIKEN, Saitama 351-0198, Japan

**Keywords:** treadmill walking, wearable sensors, gait analysis, upper trunk analysis, linear and nonlinear features, correlation analysis

## Abstract

Treadmills are widely used to recover walking function in the rehabilitation field for those patients with gait disorders. Nevertheless, the ultimate goal of walking function recovery is to walk on the ground rather than on the treadmill. This study aims to determine the effect of treadmill walking on gait and upper trunk movement characteristics using wearable sensors. Eight healthy male subjects are recruited to perform 420-m straight overground walking (OW) and 5 min treadmill walking (TW), wearing 3 inertial measurement units and a pair of insole sensors. In addition to common linear features, nonlinear features, which contains sample entropy, maximal Lyapunov exponent and fractal dynamic of stride intervals (detrended fluctuation analysis), are used to compare the difference between TW and OW condition. Canonical correlation analysis is also used to indicate the correlation between upper trunk movement characteristics and gait features in the aspects of spatiotemporal parameters and gait dynamic features. The experimental results show that the treadmill can cause a shorter stride length, less stride time and worsen long-range correlation of stride intervals. And the treadmill can significantly increase the stability for both gait and upper trunk, while it can significantly reduce gait regularity during swing phase. Canonical correlation analysis results show that treadmill can reduce the correlation between gait and upper trunk features. One possible interpretation of these results is that people tend to walk more cautiously to prevent the risk of falling and neglect the coordination between gait and upper trunk when walking on the treadmill. This study can provide fundamental insightful information about the effect of treadmill walking on gait and upper trunk to support future similar studies.

## 1. Introduction

Treadmills are widely used to recover walking function in rehabilitation field for patients with diseases of gait disorders, including Parkinson’s [1], stroke [2] and spinal cord injury [3]. Compared with training on the ground, treadmill training has superior advantages, such as accessible assistance, less space requirement, controlled gait speed [4]. Nevertheless, the ultimate goal of walking function recovery is to walk on the ground rather than the treadmill. Therefore, determining the effect of treadmill walking on human body is very important.

Previous studies have compared the differences between treadmill walking (TW) and overground walking (OW) conditions in kinematics [5,6,7,8], kinetics [8,9,10] and energy consumption [11]. These two types of walking conditions were compared from aspects of spatiotemporal characteristics, gait dynamic analysis and upper trunk analysis. Regarding spatiotemporal parameters, when the speed of TW is the same as OW, Lee et al. [4] found that young and elderly people both had a longer stance time under OW condition. And Watt et al. [12] observed that stride time and length of OW were greater than TW. For the last decade, many methods have been used to conduct gait dynamic analysis. Concerning the center of pressure (COP), Grieco et al. [13] calculated COP path efficiency and the position of cyclogram intersection point (CISP) to measure gait consistency for angelman syndrome children and healthy children. And they found a less efficient and consistent COP pathway in angelman syndrome children. Deborah et al. [14] found that COP velocity in anterior-posterior (AP) direction of healthy older adults was larger than those with Parkinson’s disease. Samira et al. [15] calculated the sample entropy of COP to compare treadmill walking only and dual-task condition. But there are only a few studies concentrating on the difference of COP features between TW and OW condition. Additionally, foot clearance is a key factor related to the risk of falling [16,17]. Dadashi et al. [18] found that foot clearance could characterize the risky gait pattern through analyzing the dataset from 1400 participants. Arami et al. [19] designed an accurate wearable foot clearance estimation system with infrared distance meter sensors and inertial measurement unit to provide a real-time estimation of foot height and orientation. Several related studies about gait analysis are presented in Table 1.

Upper trunk movement is also an important part of locomotor system when walking. Dingwell et al. [20] researched the short-term and long-term stability of upper body by mounting a tri-axial accelerometer at the base of the sternum and they found a strong effect of treadmill. Terrier et al. [21] compared the nonlinear features of upper trunk between TW and OW condition from aspects of fractal dynamic (by detrended fluctuation analysis) and local dynamic stability (by maximal Lyapunov exponent). They found that treadmill increased dynamic stability in mediolateral (ML), vertical (V) and AP directions and induced a less correlated pattern in stride intervals.

Although many studies have compared the TW and OW conditions from different aspects, the comparison is not comprehensive. For example, Samira et al. [15] recorded forty s data to analyze gait features while subjects were possibly not ready to walk normally. Terrier et al. [21], who attached a tri-axial accelerometer to subject’s trunk, just took into account the upper trunk features except gait features. Nonlinear methods for human movement analysis mainly contain entropy, Lyapunov exponent and fractal dynamic analysis. Previous studies only used part of these to compare the difference. Moreover, as a typical feature for gait analysis, few studies have focused on the difference of COP features between the two types of conditions.

The purpose of this study is to determine the effect of treadmill on both gait and upper trunk movement characteristics by using 3 inertial measurement units and a pair of insole sensors. In addition to the linear analysis methods, nonlinear methods are applied, including entropy, Lyapunov exponent and fractal dynamic analysis. Moreover, the relationship between upper trunk movement characteristics and gait features in the aspects of spatiotemporal parameters and gait dynamic features is evaluated through canonical correlation analysis.

## 2. Methods

### 2.1. Participants

Eight healthy male subjects, with no orthopedic impairment, neurological or other system disorders, are recruited to participate in this experiment. Considering effects of age and gender [22,23], their characters are (Mean ± Std): age 25 ± 3 year., body mass 67 ± 8 kg, height 1.72 ± 0.05 m. Before starting formal experiment, all subjects are required to be trained on the experimental treadmill more than 10 min for adapting to the treadmill. Eight healthy male subjects participating in this experiment read and signed an informed consent form that was approved by the Research Ethics Committee of Nankai University before starting this experiment.

### 2.2. Apparatus

The instruments used in this experiment include 3 inertial measurement units (size: 41 × 37 × 11 mm, weight: 12 g) and a pair of insole sensors (Novel, Munich, Germany). Inertial measurement unit, mainly consisting of a 9-axial sensor (MPU-9250), a microcontroller unit (STM32F103C8T6), a battery and a wireless module (nRF24L01), can acquire acceleration, angular velocity, magnetic data in ML, V and AP directions. A laptop is used to receive data from inertial measurement unit in real-time. Since the data cannot be received at a constant frequency (about 400–500 Hz) due to wireless transmission, the signal is downsampled at 200 Hz. After downsampling, the data from accelerometer and magnetometer is calibrated with the ellipsoid fitting method based on least square method. Then Madgwick’s algorithm is used to solve attitude quaternions [24]. Insole sensors can collect plantar pressure data at 100 Hz and a data logger can reserve insole sensors’ data into an SD card. COP positions data is able to be acquired through Pedar software (Novel, Munich, Germany). PyCharm (JetBrains, Prague, Czech Republic) and SPSS 25.0 (IBM, Armonk, NY, USA) are used to perform data analysis.

### 2.3. Experimental Protocol

In order to ensure that participants walk normally rather than being disturbed by this experiment, each participant is required to conduct OW test on a 420-m straight road and then perform 5 min TW test on a usual treadmill (Yijian, Hangzhou, China) without holding onto the handrails due to its possible effect for gait pattern. The speed of treadmill is set to the value obtained by OW test. It almost takes 5 min to finish walking on the 420-m straight road. Each participant is asked to walk along a straight line to exclude the influence of unrelated factors under OW test. 

**Figure 1:**
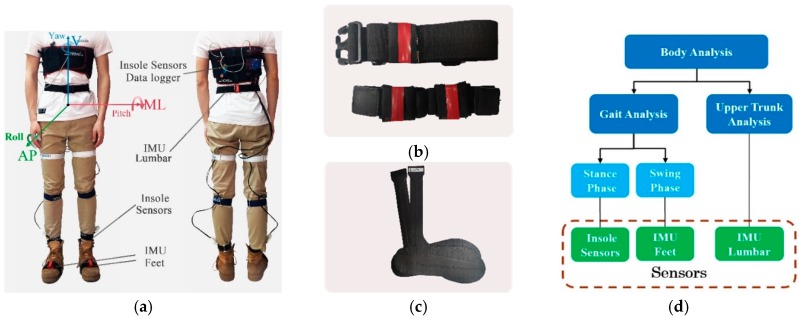
(**a**) Wearable sensors used in this experiment, including three inertial measurement unit (IMU) and a pair of insole sensors. Two IMU are attached to feet with elastic belt in the position that is closed to toe (IMU Feet). The other is attached to the low back, where it is closed to L3 region (IMU Lumbar). A pair of insole sensors are put into subject’s own shoes and the data logger of insole sensors is fastened in the subject’s body. In addition, mediolateral (ML), vertical (V), anterior-posterior (AP), Pitch, Roll and Yaw directions are shown. (**b**) Representation of three IMU with elastic belt. (**c**) A pair of insole sensors (Novel, Germany). (**d**) A diagram of human body analysis using insole sensors and IMU.

Gait and upper trunk data are both collected to comprehensively analyze the difference between two conditions. For the gait analysis, one inertial measurement unit is attached to each foot in the position that is closed to toe for recording acceleration of V direction. Acceleration data can be utilized to analyze gait regularity and stability of swing phase. Insole sensors are put into subject’s shoes to acquire plantar pressure data for gait analysis during stance phase. A data logger of insole sensors is fastened in subject’s body, as shown in Figure 1a. For the upper trunk analysis, one inertial measurement unit is attached to the lower back (L3 region) with an elastic belt. In addition, experimenter need to carry a laptop and follow the subject to record inertial measurement unit’s data in real-time.

### 2.4. Linear Features

To avoid non-stationary periods, 75 s to 275 s of measurement data are used among 5 min measurement. Regarding spatiotemporal parameters, three linear features are compared refer to previous studies, which were stride length, stride time and coefficient of variation (CV) of stride time. The CV of stride time is presented in Equation (1),

(1) $$\mathrm{CV}=\frac{\mathrm{Std}}{\mathrm{Mean}}\times 100$$

where CV is coefficient of variation, Std is the standard deviation of stride time and Mean is the average of stride time.

**Figure 2:**
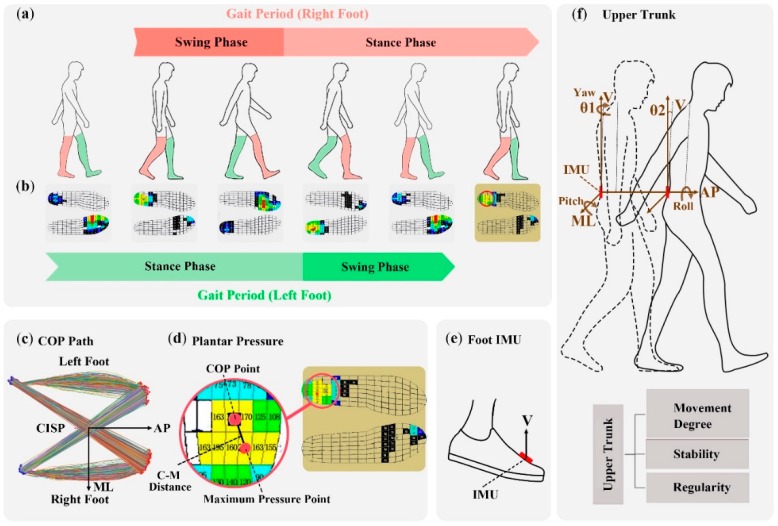
(**a**) A diagram of entire gait period for left and right foot, including swing phase and stance phase. (**b**) The Plantar pressure during entire gait period for analyzing gait features of stance phase. (**c**) Representation of center of pressure (COP) path cyclogram. CISP, ML, V and AP respectively denote cyclogram intersection point, mediolateral, vertical and anterior-posterior. During stance phase, COP position moves forward under support foot. When support foot becomes the other foot, COP position moves from one foot to the other. (**d**) The position of COP point, the position of maximum pressure point and C-M distance means the average distance between these two points. (**e**) IMU is attached to foot for analyzing gait features of swing phase. (**f**) A diagram of upper trunk movement degree, stability and regularity when walking. θ means upper trunk movement degree in pitch direction.

In order to exclude the influence of walking velocity, all RMS of acceleration in this experiment is divided by the square of gait velocity [25]. RMS of foot acceleration in V direction is calculated so as to analyze foot stability for gait analysis of swing phase [26]. Foot acceleration in ML and AP directions is not used in this experiment because treadmill can move subject’s feet under TW test. It is meaningless to compare these features under two conditions. In addition, all the acceleration data is filtered with low-pass Butterworth filter (4 order, 3 Hz, zero-phase filtering) before calculating RMS of acceleration. For gait analysis of stance phase, four linear features are adopted, containing COP path efficiency, the standard deviation of ML and AP positions of cyclogram intersection point (Std ML-CISP, Std AP-CISP), the average distance between COP point and maximum pressure point (C-M distance). The COP path efficiency is calculated through dividing direct COP distance by the actual path that COP travelled during stance phase. It signifies how the COP point traveled position from the beginning to the end of the stance phase. During stance phase, COP position moves forward under support foot. When support foot becomes the other foot, COP position moves from one foot to the other. CISP means the point where COP path is crossing by itself [13]. Std ML-CISP and Std AP-CISP can measure the inconsistency of COP pathway during stance phase, as shown in Figure 2c. Apart from COP position, the average C-M distance that represents the degree of uniform distribution of foot pressure during stance phase is calculated, as shown in Figure 2d.

Concerning the upper trunk analysis, root mean square (RMS) of movement degree in pitch, roll and yaw directions are computed based on inertial measurement units’ data. RMS of lumbar acceleration in ML, V and AP directions can denote upper trunk instability and a larger value indicates poorer stability. All linear features used in this study are shown in Table 2.

### 2.5. Nonlinear Features

Nonlinear mathematical analysis can describe complex conditions of human movement in which linear techniques are inadequate [27]. Three nonlinear analysis methods are used for gait and upper trunk analysis in this experiment, including entropy, Lyapunov exponent and fractal dynamic. When analyzing nonlinear features, filtering is avoided to preserve intact information of nonlinear dynamics.

#### 2.5.1. Sample Entropy

Entropy can quantitatively measure the irregularity or unpredictability of human movement. Several different methods have been proposed to calculate the entropy of time series, mainly containing approximate entropy, sample entropy, symbolic entropy and multi-scale entropy [27]. Among them, sample entropy is one of the most popular methods to research human movement because of less sensibility to changes in data length [28]. The calculation of sample entropy is shown as follows:

(2) $$B=\frac{1}{N-m}\sum_{i=1}^{N-m}B_{i}^{m},$$

(3) $$A=\frac{1}{N-m-1}\sum_{i=1}^{N-m-1}A_{i}^{m+1},$$

(4) $$\mathrm{SE}=-\ln\frac{A}{B},$$

where *m* is vector length, *N* is the length of entire time serious and SE is sample entropy. Vector length *m* is used to divide the entire time series of length *N* into short vectors of length *m* and *m* + 1. $B_{i}^{m}$ denotes the probability of finding vectors similar to the *i*-th vector among all the short vectors of length *m* below the tolerance radius *r*. The sum of conditional probability for *m* and *m* + 1 divided by N−m were defined as *B* and *A*, then sample entropy was computed by Equation (4).

Choosing approximate vector length *m* and tolerance radius *r* are important to calculate sample entropy. According to previous papers reporting sample entropy [29,30], vector length *m* is set to 2 and tolerance *r* is set to 0.2 times standard deviation of the original data. In this experiment, sample entropy is used for gait and upper trunk analysis, specifically containing foot acceleration during swing phase in V direction, COP position in ML and AP directions and lumbar acceleration in ML, V and AP directions.

#### 2.5.2. Maximal Lyapunov Exponent

Calculation of maximal Lyapunov exponent λ is a popular method to quantify dynamical stability of human movement [31]. It quantifies the average rates of divergence or convergence of nearby trajectories in state space and the larger it is, the more unstable the original data is. Wolf et al. algorithm [32] and Rosenstein et al. algorithm [33] are the two most widely used algorithms to compute Lyapunov exponent. Compared to Wolf et al. algorithm, Rosenstein et al. algorithm is more robust with short and noisy data, and therefore it is used in this experiment. Embedding dimension *m* and time lag *T* are important parameters when computing Lyapunov exponent λ. The detailed computation of λ is shown in the following equations:

(5) $$X(t)=[x(t_{0}),x(t_{0}+T),x(t_{0}+2T),\cdots ,x(t_{0}+(m-1)T)],$$

(6) $$d_{i}(t)\approx e^{\lambda_{i}t}d_{i}(t_{0}),\;\ln d_{i}(t)=\lambda_{i}t+\ln d_{i}(t_{0}),$$

where X(t) is delay vector that has *m* elements. $x(t_{0})$ is the initial point and $t_{0}$ is set from 0 to N−(m−1)T. $d_{i}(t_{0})$ is initial divergence and the divergence value becomes $d_{i}(t)$ after time *t*. Embedding dimension *m* and time lag *T* are used to generate N−(m−1)T delay vectors, and the distance $d_{i}(t)$ between these two delay vectors on the trajectories at each step is computed. According to Equation (6), the maximal Lyapunov exponent λ is the greatest slope when plotting $\ln d_{i}(t)$ versus *t*. Rosenstein et al. suggested to set time lag *T* to the autocorrelation function drops below 1–1/*e* times its original value. Embedding dimension *m* is set to 6 computed from false nearest neighbor algorithm in this experiment [34]. All features that are used with sample entropy also are analyzed with the maximal Lyapunov exponent to measure dynamic stability for both gait and upper trunk analysis.

#### 2.5.3. Fractal Dynamic

The DFA method can measure stride intervals’ correlation in a long time series, which is called fractal dynamic. It is a monofractal method based on classic root mean square analysis [27]. The calculation of this method is described as follows:

(7) $$y(k)=\sum_{i=1}^{k}\left[y(i)-\bar{y}\right],k=1,2,\cdots ,N$$

(8) $$D_{n}(k)=y(k)-y_{n}(k),$$

(9) $$F(n)=\sqrt{\frac{1}{N}\sum_{k=1}^{N}{\left[D_{n}(k)\right]}^{2}},$$

where *N* is the length of original time series and y is the data of time series. The original time series of length *N* is integrated from its mean value $\bar{y}$ to get y(k). For different box size *n*, $y_{n}(k)$ is fit to the segment in each box, then y(k) is detrended through subtracting local trend $y_{n}(k)$ to get $D_{n}(k)$. Magnitude of fluctuation $F_{n}$ is calculated by root mean square computation for each box size *n*. Scaling exponent α is the slope when plotting the line between $\log F_{n}$ and logn.

Scaling exponent α of stride intervals signifies the fluctuation of stride intervals. When α is between 0.5 and 1, the time series of stride intervals is thought to have long-range correlation [27]. In addition, scaling exponent α under two walking conditions is compared to reveal the effect of treadmill walking on fractal dynamic. All nonlinear features used in this study are shown in Table 3.

### 2.6. Statistical Analysis

Paired t-tests are performed to compare linear and nonlinear features between TW and OW condition. A 95% confidence interval was computed as mean value ± 1.96 times the standard error of mean (SEM, N = 8). Effect size is given as the difference of mean value for each paired parameter and it is normalized by Hedges′g due to its small sample sizes (N < 20). Following the rule thumb suggested by Cohen, the value of 0.2, 0.5, and 0.8 respectively represents small, medium and large effects [35]. Canonical correlation analysis is performed to indicate the correlation between gait and upper trunk features.

## 3. Results

### 3.1. Gait Analysis Result

Table 4 presents comparison of gait spatiotemporal parameters between TW and OW condition. Statistical results show that people have a greater stride length (OW: 1.391 ± 0.112, TW: 1.341 ± 0.103 m, p = 0.028) and longer stride time (OW: 1.062 ± 0.049, TW: 1.031 ± 0.052, p < 0.01) under OW condition. Namely, people spend less time finishing an intact gait period (higher cadence) with a shorter stride length when walking on the treadmill. CV of stride time can measure gait variability in stride time and there is no obvious difference between two conditions. From scaling exponent α of stride intervals, TW and OW condition both have long-range correlation in stride intervals (OW: 0.819 ± 0.150, TW: 0.663 ± 0.101), and treadmill significantly changes the fractal dynamic (p = 0.016).

RMS, sample entropy and the maximal Lyapunov exponent of acceleration in V direction are used to conduct gait analysis of swing phase. From Table 5, there is a greater RMS value (OW: 2.021 ± 0.438, TW: 1.534 ± 0.392 m/s^2^, p < 0.01) and lower sample entropy (OW: 0.115 ± 0.016, TW: 0.341 ± 0.045, p < 0.01) under OW condition, which reveals that treadmill can bring better stability and worse regularity for gait during swing phase, while there is no difference in the maximal Lyapunov exponent under the two conditions.

Gait analysis of stance phase is conducted with COP features. From Table 6, there is a higher COP path efficiency (OW: 96.406% ± 0.980%, TW: 95.337% ± 1.811%, p = 0.029) and lower stability of AP direction (OW: −0.006 ± 0.012, TW: -0.018 ± 0.016, p = 0.042) under OW condition. During the stance phase, no difference is shown in C-M distance, Std ML-CISP, Std AP-CISP, sample entropy of ML and AP directions and stability of ML direction. The results of the COP features analysis indicate that treadmill does not alter distribution of foot pressure, COP path consistency and regularity during stance phase, while it increases the stability of AP direction and reduces COP path efficiency.

### 3.2. Upper Trunk Analysis Result

From Table 7, there is no significant difference on upper trunk movement degree and regularity in pitch, roll and yaw directions under two conditions. RMS of lumbar acceleration and maximal Lyapunov exponent can both reflect the stability of upper trunk. Taking these two features into account, the treadmill can increase upper trunk stability due to RMS of lumbar acceleration in V direction (OW: 0.840 ± 0.212, TW: 0.650 ± 0.216 m/s^2^, p < 0.01) and AP direction (OW: 1.267 ± 0.182, TW: 1.059 ± 0.243 m/s^2^, p = 0.014), and the maximal Lyapunov exponent in ML direction (OW: 0.044 ± 0.013, TW: 0.026 ± 0.011, p = 0.043).

λ signifies the maximal Lyapunov exponent computed by Rosenstein et al. algorithm. Mediolateral (ML), vertical (V), anterior-posterior (AP), pitch, roll and yaw directions are presented in Figure 1a. C-M distance that is shown in Figure 2d means the average distance between COP point and maximum pressure point. Std ML-CISP and Std AP-CISP respectively represents the standard deviation of cyclogram intersection point position in ML and AP directions, as shown in Figure 2c. The effect size value can measure the normal difference by OW − TW and the normalized difference by Hedges′g. T-test column shows the p value of paired t-tests between TW and OW condition and significant p-values (p < 0.05) are denoted in bold. All the value is saved in three decimals by rounding off. The calculation of CI and Hedges′g is shown as follows:

(10) $$\mathrm{CI}=\mathrm{Mean}\pm 1.96\times \mathrm{SEM},\;\mathrm{SEM}=\frac{Std}{\sqrt{n}},$$

(11) $$Hedges’g=\frac{M_{1}-M_{2}}{Std_{\mathrm{polled}}^{}},$$

(12) $$Std_{\mathrm{polled}}^{}=\sqrt{\frac{(n_{1}-1)Std_{1}^{2}+(n_{2}-1)Std_{2}^{2}}{n_{1}{+n}_{2}-2}},$$

where CI means confidence interval computed as mean value ± 1.96 times the standard error of mean (SEM, n = 8). $M_{1},M_{2}$ denote the mean value and $Std_{1},\;Std_{2}$ denote standard deviation under TW and OW condition respectively. In this experiment, n means the number of subjects under two conditions (${n=n}_{1}{=n}_{2}=8$).

## 4. Discussion

### 4.1. Difference between Treadmill and Overground Walking

**Figure 3:**
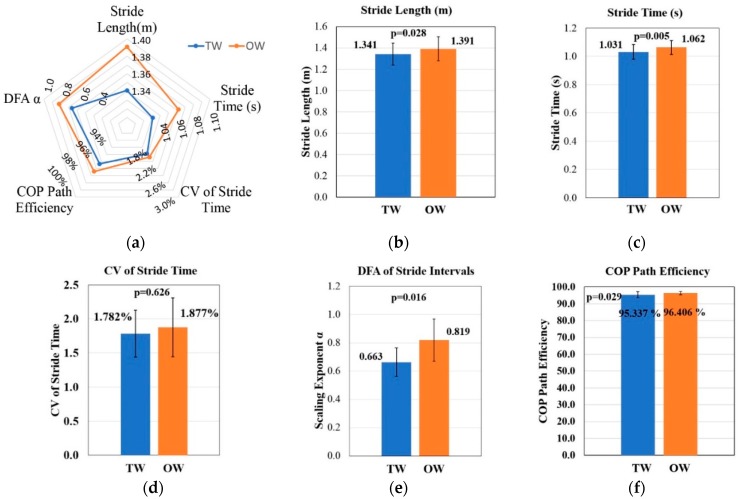
(**a**) Contrast of spatiotemporal parameters between TW and OW condition. (**b**) Contrast of stride length. (**c**) Contrast of average stride time. (**d**) Contrast of CV of stride time. (**e**) Contrast of fractal dynamic of stride intervals. (**f**) Contrast of COP path efficiency.

In the above, we have thoroughly compared the difference between TW and OW condition for both gait and upper trunk features. As shown in Figure 3, spatiotemporal parameters are different in stride length, stride time and scaling exponent α of stride intervals though people walk at the same speed under two conditions. Scaling exponent α of stride intervals reflects the effect of walking condition on fractal dynamic and it can be used to analyze the feedback characteristics in motor control. Experimental results show that treadmill significantly reduces the long-range correlation of stride intervals. In previous studies, Watt et al. [11] also observed that stride time and stride length were greater under OW condition when walking at the same speed under two conditions. Terrier et al. [20] also found that treadmill can cause the decrease of long-range correlation in stride intervals. Although stride time and fractal dynamic of stride intervals under two conditions are different, CV of stride time is very similar. The treadmill does not alter gait variability in terms of stride time. While it can reduce COP path efficiency, which means that the COP position moves in a more winding path during stance phase when walking on the treadmill.

**Figure 4:**
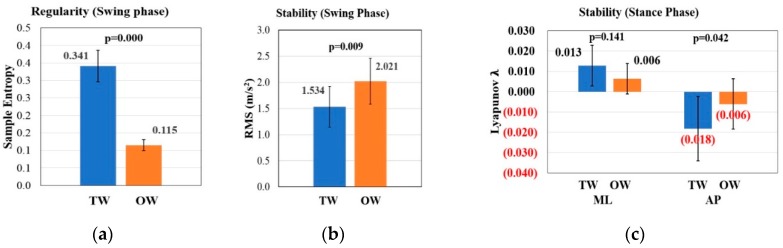
Comparison of gait features between TW and OW condition: (**a**) Sample entropy of foot acceleration during swing phase in V direction. (**b**) The RMS of foot acceleration during swing phase in V direction. (**c**) The maximal Lyapunov exponent of COP position during stance phase in ML and AP directions.

When conducting gait analysis, gait period is divided into swing phase and stance phase. As shown in Figure 4, during swing phase, treadmill can significantly cause more irregularity in V direction while it can increase the stability in V direction. During stance phase, treadmill also increases the stability in AP direction.

**Figure 5:**
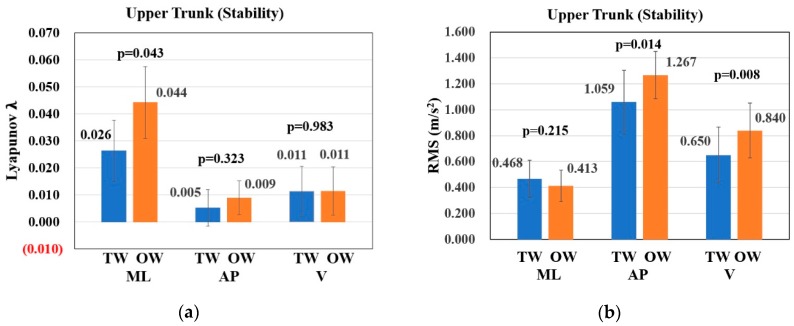
Comparison of upper trunk stability between TW and OW condition: (**a**) The maximal Lyapunov exponent of lumbar acceleration in ML, V and AP directions. (**b**) The RMS of lumbar acceleration in ML, V and AP directions.

Under two conditions (as shown in Figure 5), the movement degree and regularity of upper trunk are very similar. It means that the treadmill does not change the kinematic characteristics of upper trunk. Considering both the RMS of acceleration and maximal Lyapunov exponent, treadmill significantly increases stability of upper trunk in ML, V and AP directions. One possible interpretation is that people try to be more careful and focus on their body stability in case of falling when walking on the treadmill. This behavior can result in the increase of stability for both gait and upper trunk, and it can change some spatiotemporal parameters.

### 4.2. Correlation between Gait and Upper Trunk Features

Although the gait and upper trunk features have been analyzed independently under both OW and TW condition, it is possible that the relationship exists between gait and upper trunk features. The relationship can reflect how people coordinate their gait and upper trunk when walking, and the treadmill can possibly affect the coordination. Canonical correlation analysis is conducted to evaluate the relationship between gait features and upper trunk features. This method can indicate the correlations through Pearson’s correlation coefficients. Table 8 shows the gait and upper trunk features that have a significant correlation. Under OW condition, walking speed has a significantly negative correlation with the movement degree, regularity and stability of upper trunk, while stride time has a positive correlation with the movement degree and regularity of upper trunk. Moreover, COP path efficiency has a significantly negative correlation with the movement degree and stability of upper trunk. And a negative correlation exists between gait stability in ML direction and upper trunk regularity. However, all the correlations between gait and upper trunk features decrease or disappear except the relationship between walking speed and the movement degree of upper trunk when walking on the treadmill. People tend to walk more cautiously, but ignore the coordination of their locomotor system on the treadmill; this will lead to the increase of stability for both gait and upper trunk and the decrease of correlation between gait and upper trunk features.

### 4.3. Technical Issues

There are many sensors used in analysis of human movement including wearable sensors, floor sensors and image sensors [36]. For the present study, portable inertial measurement unit and insole sensors are used to acquire experimental data because they can record long-distance and long-term walking rather than walking in restrictive space. Inertial measurement units are attached to the lower back and feet to collect gait and upper trunk movement data and insole sensors can assess force when walking.

Walking speed is a vital factor to research the effect of treadmill on human movement. According to previous studies, there are three different ways to set the treadmill’s speed. The first one is to set treadmill’s speed to the value obtained under OW test [4]. The second one is to adopt the same treadmill’s speed for all subjects, obtained by assessing the average preferred walking speed on the same treadmill [21]. The last way of determining treadmill’s speed is to ask subjects to walk with their own preferred walking speed on the treadmill [37]. In order to exclude the influence of walking speed, the first way was used in this experiment.

Different types of shoes are possible to affect gait and upper trunk movement when walking. For example, Davis et al. reported that footwear with dorsal fixation could bring greater minimum foot clearance than the slippers and bare feet in older women [38]. Hong et al. found that ankle-covered shoes had a positive influence on the gait of stroke survivors [39]. In our experiment, subjects are required to wear their own comfortable shoes and each subject finishes walking on the treadmill and the ground with the same shoes.

## 5. Conclusions

In this study, we investigated the effect of treadmill walking on gait and upper trunk by using wearable sensors. Linear and nonlinear analysis methods were used to evaluate the changes of spatiotemporal parameters, regularity and stability for both gait and upper trunk when walking on the treadmill. The results of this study can provide fundamental information to future similar studies, because gait analysis and walking function recovery on the treadmill are both based on the hypothesis that TW is equivalent to OW. A limitation of this study is that different subjects possibly have different degree of adaptation to the treadmill although all of them are required to be trained on the treadmill more than 10 min, which can cause individual variation of experimental results. In the future study, we will apply the linear and nonlinear analysis methods to track motion stability of elderly people and can provide some guidance for elderly people in exercise.

## Figures and Tables

**Table 1:** Several related studies about gait analysis.

Gait Variable	Authors	Results	References
**Spatiotemporal parameters**	Lee et al.	Young and elderly people both had longer stance time under OW condition.	[4]
	Watt et al.	Stride time and length of OW were greater than TW.	[12]
**COP**	Grieco et al.	A less efficient and consistent COP pathway was shown in angelman syndrome children than healthy children.	[13]
	Deborah et al.	COP velocity in anterior-posterior direction of healthy older adults was larger than those with Parkinson diseases.	[14]
	Samira et al.	Sample entropy of COP could maintain the directional difference between treadmill walking only and dual-task condition across variant parameter values.	[15]
**Foot clearance**	Dadashi et al.	Foot clearance could characterize the risky gait pattern through analyzing the dataset from 1400 participants.	[18]
	Arami et al.	An accurate wearable foot clearance estimation system with infrared distance meter sensors and inertial measurement unit was designed to provide a real-time estimation of foot height and orientation.	[19]

**Table 2:** Linear features used in this study and the data source used to calculate features.

Linear Features		Data Source
**Spatiotemporal parameters**	Stride length	Insole sensors
	Stride time	Insole sensors
	CV of stride time	Insole sensors
**Gait features of swing phase**	RMS of acceleration in V direction	IMU Feet
**Gait features of stance phase (COP)**	COP efficiency	Insole sensors
	C-M distance	Insole sensors
	Std ML-CISP	Insole sensors
	Std AP-CISP	Insole sensors
**Upper trunk features**	RMS of movement degree in pitch direction	IMU Lumbar
	RMS of movement degree in roll direction	IMU Lumbar
	RMS of movement degree in yaw direction	IMU Lumbar
	RMS of acceleration in ML direction	IMU Lumbar
	RMS of acceleration in V direction	IMU Lumbar
	RMS of acceleration in AP direction	IMU Lumbar

Std ML-CISP and Std AP-CISP respectively represents the standard deviation of cyclogram intersection point position in ML and AP directions, as shown in Figure 2c. Data source including insole sensors, IMU feet and IMU lumbar, corresponds to Figure 1a.

**Table 3:** Nonlinear features used in this study and the data source to calculate features.

Nonlinear Features		Data Source
**Spatiotemporal parameters**	Scaling exponent α of stride intervals (DFA)	Insole sensors
**Gait features of swing phase**	Maximal Lyapunov exponent λ of foot acceleration in V direction	IMU Feet
	Sample entropy of foot acceleration in V direction	IMU Feet
**Gait features of stance phase (COP)**	Maximal Lyapunov exponent λ of COP position in ML direction	Insole sensors
	Maximal Lyapunov exponent λ of COP position in AP direction	Insole sensors
	Sample entropy of COP position in ML direction	Insole sensors
	Sample entropy of COP position in AP direction	Insole sensors
**Upper trunk features**	Maximal Lyapunov exponent λ of lumbar acceleration in ML direction	IMU Lumbar
	Maximal Lyapunov exponent λ of lumbar acceleration in V direction	IMU Lumbar
	Maximal Lyapunov exponent λ of lumbar acceleration in AP direction	IMU Lumbar
**Upper trunk features**	Sample entropy of lumbar acceleration in ML direction	IMU Lumbar
	Sample entropy of lumbar acceleration in V direction	IMU Lumbar
	Sample entropy of lumbar acceleration in AP direction	IMU Lumbar

**Table 4:** Comparison of spatiotemporal parameters between TW and OW condition.

	OW		TW		Effect Size		T-test
N = 8	Mean ± Std	CI	Mean ± Std	CI	OW − TW	Norm.	p
**Stride length (m)**	1.391 ± 0.112	1.313 − 1.469	1.341 ± 0.103	1.270 − 1.412	0.050	0.464	**0.028**
**Stride time (s)**	1.062 ± 0.049	1.028 − 1.096	1.031 ± 0.052	0.994 − 1.067	0.032	0.626	**0.005**
**CV of stride time (%)**	1.877 ± 0.432	1.577 − 2.177	1.782 ± 0.344	1.544 − 2.021	0.095	0.242	0.626
**α (DFA)**	0.819 ± 0.150	0.716 − 0.923	0.663 ± 0.101	0.593 − 0.733	0.156	1.223	**0.016**

**Table 5:** Comparison of gait features during swing phase in V direction between TW and OW condition.

	OW		TW		Effect Size		T-test
N = 8	Mean ± Std	CI	Mean ± Std	CI	OW − TW	Norm.	p
**RMS of acceleration**	2.021 ± 0.438	1.717 − 2.324	1.534 ± 0.392	1.262 − 1.805	0.487	1.172	**0.009**
**λ**	0.024 ± 0.005	0.021 − 0.028	0.023 ± 0.005	0.019 − 0.026	0.002	0.346	0.495
**Sample entropy**	0.115 ± 0.016	0.104 − 0.126	0.341 ± 0.045	0.310 − 0.372	−0.226	−6.715	**0.000**

**Table 6:** Comparison of gait’s COP features during stance phase in ML and AP directions between TW and OW condition.

	OW		TW		Effect Size		T-test
N = 8	Mean ± Std	CI	Mean ± Std	CI	OW − TW	Norm.	p
**COP efficiency (%)**	96.406 ± 0.980	95.726 − 97.085	95.337 ± 1.811	94.083 − 96.592	1.068	0.734	**0.029**
**C-M distance (mm)**	34.125 ±7.453	28.960 − 39.290	31.375 ± 4.658	28.147 − 34.603	2.750	0.442	0.332
**Std ML-CISP (mm)**	1.508 ± 0.318	1.287 − 1.728	1.481 ± 0.350	1.238 − 1.723	0.027	0.080	0.835
**Std AP-CISP (mm)**	3.207 ± 0.409	2.923 − 3.490	2.994 ± 0.487	2.656 − 3.332	0.213	0.473	0.211
**λ-ML**	0.006 ± 0.007	0.001 − 0.012	0.013 ± 0.010	0.006 − 0.020	−0.006	−0.733	0.141
**λ-AP**	−0.006 ± 0.012	−0.015 − 0.002	−0.018 ± 0.016	−0.029 − −0.007	0.012	0.858	**0.042**
**Sample entropy-ML**	0.062 ± 0.006	0.058 − 0.066	0.061 ± 0.006	0.057 − 0.065	0.001	0.145	0.585
**Sample entropy-AP**	0.064 ± 0.006	0.060 − 0.069	0.061 ± 0.006	0.057 − 0.065	0.003	0.474	0.387

**Table 7:** Comparison of upper trunk features between TW and OW condition.

	OW		TW		Effect Size		T-test
N = 8	Mean ± Std	CI	Mean ± Std	CI	OW − TW	Norm.	p
**RMS of pitch degree (°)**	0.909 ± 0.308	0.696 − 1.123	0.936 ± 0.243	0.767 − 1.104	−0.026	−0.095	0.623
**RMS of roll degree (°)**	0.947 ± 0.421	0.655 − 1.238	0.920 ± 0.207	0.776 − 1.063	0.027	0.082	0.785
**RMS of yaw degree (°)**	1.649 ± 0.401	1.370 − 1.927	1.764 ± 0.478	1.433 − 2.095	−0.116	−0.263	0.339
**RMS of acceleration (ML)**	0.413 ± 0.122	0.328 − 0.498	0.468 ± 0.144	0.369 − 0.568	−0.056	−0.416	0.215
**RMS of acceleration (V)**	0.840 ± 0.212	0.693 − 0.987	0.650 ± 0.216	0.500 − 0.799	0.190	0.887	**0.008**
**RMS of acceleration (AP)**	1.267 ± 0.182	1.140 − 1.393	1.059 ± 0.243	0.891 − 1.228	0.207	0.965	**0.014**
**λ-ML**	0.044 ± 0.013	0.035 − 0.053	0.026 ± 0.011	0.019 − 0.034	0.018	1.448	**0.043**
**λ-V**	0.011 ± 0.009	0.005 − 0.018	0.011 ± 0.009	0.005 − 0.018	0.000	0.010	0.983
**λ-AP**	0.009 ± 0.006	0.005 − 0.013	0.005 ± 0.007	0.001 − 0.010	0.004	0.576	0.323
**Sample entropy-ML**	0.581 ± 0.151	0.476 − 0.686	0.689 ± 0.219	0.537 − 0.841	−0.107	−0.571	0.313
**Sample entropy-V**	0.376 ± 0.111	0.299 − 0.453	0.515 ± 0.141	0.418 − 0.613	−0.139	−1.098	0.086
**Sample entropy-AP**	0.390 ± 0.045	0.358 − 0.421	0.401 ± 0.078	0.347 − 0.455	−0.012	−0.181	0.759

**Table 8:** Pearson’s correlation matrix between gait features and upper trunk features under OW and TW conditions.

N = 8		Gait							
		Walking Speed		Stride Time		COP Path Efficiency		λ-ML	
		OW	TW	OW	TW	OW	TW	OW	TW
**Upper Trunk**	**RMS of pitch degree**	−0.313	−0.225	0.295	0.234	−0.779 *	−0.152	0.212	0.054
	**RMS of roll degree**	−0.881 **	−0.667	0.813 *	0.450	−0.754 *	−0.457	−0.475	0.383
	**RMS of yaw degree**	−0.452	−0.733 *	0.648	0.578	−0.323	−0.659	−0.334	0.539
	**Sample entropy-ML**	−0.659	−0.191	0.900 **	0.044	−0.379	−0.390	−0.718 *	0.417
	**Sample entropy-V**	−0.549	0.012	0.835 **	−0.460	−0.164	0.050	−0.813*	0.346
	**Sample entropy-AP**	−0.882 **	0.052	0.744 *	−0.306	−0.571	−0.098	−0.427	0.184
	**RMS of acceleration-ML**	−0.841 **	−0.604	0.632	0.353	−0.606	−0.242	−0.290	0.183
	**RMS of acceleration-V**	−0.852 **	−0.680	0.600	0.672	−0.787 *	−0.670	−0.162	0.366
	**RMS of acceleration-AP**	−0.472	−0.197	0.242	0.221	0.047	−0.165	−0.244	0.178

Bold printed * represents significant correlation at 0.05 level (2-tailed) and bold printed, ** represents significant correlation at 0.01 level.
